# Physical Fitness Variables, General Health, Dementia and Quality of Life in Individuals with Intellectual and Developmental Disabilities: A Cross-Sectional Study

**DOI:** 10.3390/healthcare11192688

**Published:** 2023-10-06

**Authors:** Miguel Jacinto, Rui Matos, Beatriz Gomes, André Caseiro, Raul Antunes, Diogo Monteiro, José Pedro Ferreira, Maria João Campos

**Affiliations:** 1 Faculty of Sport Sciences and Physical Education, University of Coimbra, 3040-248 Coimbra, Portugal; beatrizgomes@fcdef.uc.pt (B.G.); afgcaseiro@gmail.com (A.C.); jpferreira@fcdef.uc.pt (J.P.F.); mjcampos@fcdef.uc.pt (M.J.C.); 2 Life Quality Research Centre (CIEQV), 2040-413 Rio Maior, Portugal; rui.matos@ipleiria.pt (R.M.); raul.antunes@ipleiria.pt (R.A.); diogo.monteiro@ipleiria.pt (D.M.); 3 ESECS—Polytechnic of Leiria, 2411-901 Leiria, Portugal; 4 Research Center for Sport and Physical Activity (CIDAF), 3040-248 Coimbra, Portugal; 5 Center for Innovative Care and Health Technology (ciTechCare), Polytechnic of Leiria, 2411-901 Leiria, Portugal; 6 Portugal Research Center in Sport Sciences, Health Sciences and Human Development (CIDESD), 5001-801 Vila Real, Portugal

**Keywords:** anamneses, assessment, cardiovascular disease, intellectual disability, metabolic disease

## Abstract

The average life expectancy of individuals with intellectual and developmental disabilities (IDDs) is increasing. However, living more years does not mean living better, leading to the need for research on comorbidities associated with the aging process. Associated with this process are the physical characteristics most prevalent in an individual with IDD: low levels of all physical capacities, the accumulation of central fat, hyperglycemia, dyslipidemia, and hypertension, variables considered to be some of the main risk factors of the onset of metabolic and cardiovascular diseases, and variables that can negatively impact quality of life (QoL). Therefore, the aim of this study is to evaluate a sample of 21 institutionalized adults with IDD (42.81 ± 10.99 years old) in terms of their anthropometric characteristics, body composition, general health status, functional capacity, neuromuscular capacity, and dementia/cognitive function, and the possible associations with QoL. All assessments were performed in the laboratory of the Faculty of Sport Sciences and Physical Education—University of Coimbra. Participants, in the present study, have low levels of physical fitness and high metabolic and cardiovascular markets, which need to be improved. On the other hand, functional and neuromuscular ability seems to be associated with QoL (*p* ≤ 0.05). This study highlights the role of primary and secondary care providers in diagnosis, prevention, and supporting individuals with IDDs to promote QoL.

## 1. Introduction

Individuals with intellectual and developmental disabilities (IDDs) are characterized by a deficit in intellectual and adaptive functioning in the conceptual, social, and practical domains, and can be identified with mild, moderate, severe and profound degrees of disability developed before the age of 22 [1]. Around 17.79% of Portuguese citizens over the age of five reported having major or total difficulties in performing at least one of the six activities of daily living (seeing/sight, hearing, walking, memory/concentration, bathing/dressing, and understanding/making oneself understood) [2].

The accumulation of central fat, hyperglycemia, dyslipidemia, and hypertension are some of the main risk factors for the onset of metabolic diseases and cardiovascular diseases [3,4], and are considered some of the main causes of death in individuals with IDD. When analyzing the population with disabilities (motor, intellectual, or sensory), the prevalence of these factors triples compared to that in the general population [5,6].

Greater longevity is associated with a higher prevalence of cardiovascular and metabolic diseases and other comorbidities [7]. Between 1996 and 2001, the age of death for individuals with IDD was 45 years old (similar for men and women) [8]. From 2003 to 2012, the average age of death increased to 55 years old [9]. Therefore, there has been an increase in the average life expectancy of this population over the years [10], explaining the greater need to study the effects of intervention strategies that promote health improvement and reduce the impact of comorbidities associated with aging [11]. The most common symptoms of aging in individuals with intellectual disabilities include confusion, difficulty with problem resolution, communication, and socialization. It is important that individuals receive appropriate services (educational support, behavioral intervention, vocational training, family education, government resources, and psychopharmacologic interventions), reducing the risk of adverse health conditions, in order to maintain/promote their independence and quality of life (QoL) as they age [12].

Individuals with IDD face inequalities in access to health care [13,14]. These individuals may have significantly different medical, social, emotional, and educational needs compared to those of individuals without disabilities. Similarly, they may often lack the capacity to perceive, report, or address symptoms of disease, which can delay detection and intervention [15,16]. On the other hand, any of the health problems associated with the premature aging of individuals with IDDs begin around the fifth decade of life [17].

These health problems are also attributed to their sedentary lifestyle, and physical inactivity, which are associated with various factors such as a possible lack of motivation, barriers to physical activity, and an unhealthy diet [18,19]. These factors are associated with low physical fitness and health problems such as osteoporosis, musculoskeletal disorders, dementia, and metabolic and cardiovascular disease [20,21]. Similarly to the case for a population without disabilities, a sedentary lifestyle [22], physical inactivity [23], and a higher incidence of obesity [6,24] lead to a major decrease in physical fitness [25,26]. In addition, sedentary lifestyles and low adherence to physical activity also lead these individuals to have low levels of physical abilities.

Considering that the population with IDDs can present all the aforementioned comorbidities and, therefore, negatively influence the activities of daily living and QoL [27,28,29], constant monitoring and the definition of strategies that can promote these variables have become urgent.

Schalock et al. [30] refers to QoL as a set of factors that address an individual’s well-being or the perception of their social position, in the context and culture in which they are positioned, comprising sociocultural values, needs, expectations, and individual preferences. It is a multidimensional phenomenon composed of factors and domains, influenced by personal characteristics and environmental contexts.

Primary and secondary care agents should be able to diagnose, treat, and support individuals with IDD in the promotion of QoL. Identifying individuals with risk factors and developing specific interventions is urgent. It is hoped that early detection and the appropriate treatment/promotion of problems by primary and secondary care agents will help individuals with IDD to maximize their potential and integration into society and promote their QoL.

Therefore, the aims of our study are to describe and characterize participants with IDD (and to understand if there are differences between them and between sexes) in the following parameters: anthropometric characteristics, body composition, general health status, functional capacity, neuromuscular capacity, and dementia/cognitive function. This evaluation will allow us to perform an initial analysis of the participants and identify some types of disease [31]. The secondary objective is to analyze the influence of these variables on the QoL of individuals with IDD. It is important to study the association of these variables with their QoL, for the development of effective strategies to promote these variables.

## 2. Materials and Methods

The study was approved by the ethics committee (after the submission of the protocol and expert evaluation) of the University of Coimbra, Faculty of Sport Sciences and Physical Education, under the code CE/FCDEF-UC/00872021, and was carried out in accordance with the Declaration of Helsinki for human studies [32]. It is an original part of a previously published study [33]. Participants/family members/guardians signed an informed consent form.

### 2.1. Participants

The participants included 21 individuals with IDD (23 to 58 years; 42.81 ± 10.99 years old), comprising 11 males (52%; 42.18 ± 10.15 years old) and 10 females (48%; 43.5 ± 12.376 years old). All individuals are institutionalized in a support institution, located in Leiria (Portugal) and are diagnosed with IDD (mild to severe).

The ensuing inclusion requirements were established: (1) age over 18; (2) mild, moderate, or severe IDD (including Down syndrome); (3) the ability to carry out movements, particularly pulling and/or pushing; (4) success in performing the assessments. The exclusion criteria included the following: (1) contraindications to exercise (e.g., high blood pressure); (2) other associated pathologies; (3) an inability to walk unaided; (4) profound IDD; (5) an inability to communicate; (6) a lack of informed consent.

### 2.2. Procedure

All assessments were carried out in the morning period, in the laboratory of the Faculty of Sport Sciences and Physical Education, University of Coimbra, by the same researchers, to reduce potential measurement errors. The data collected from all the evaluations were transcribed into a specific record form, ensuring that all ethical procedures were followed. To ensure the participants’ safety and comfort, all pre-test instructions were given. The procedures used in the present study are described in more detail in a previously published protocol [33].

#### 2.2.1. Anthropometric and Body Composition Assessment

Weight and height were determined using a scale and stadiometer (SECA 870, Hamburg, Germany). Participants were asked to wear light clothing, wear no shoes, and stand in the Frankfurt horizontal position. The standard formula for calculating body mass index (BMI) was used (BMI = body mass (kg)/height (m)^2^). Participants were divided into four categories based on the World Health Organization (WHO) classification: “underweight”, “normal weight”, “overweight”, and “obesity” [34]. Then, a body composition assessment was conducted with a tetrapolar multifrequency InBody 770 (Seoul, Republic of Korea) instrument following the manufacturer’s instructions and comprehensive guidelines described [35]. The following variables were assessed: BMI, waist circumference, total body water (L), proteins (kg), minerals (kg), fat mass (kg), muscle mass (kg), lean mass right and left arm (kg), lean trunk mass (kg), lean mass right and left leg (kg), fat right and left arm (kg), trunk fat (kg), fat right and left leg (kg) intracellular water (L), extracellular water (L), and phase angle. For anthropometric and body composition assessments, participants were encouraged not to drink caffeine or alcohol within 12 h prior to the assessments.

#### 2.2.2. General Health Status Assessment

Hemodynamic parameters, such as resting blood pressure (systolic and diastolic) and resting heart rate were measured using a digital sphygmomanometer, Omron Digital Blood Pressure Monitor HEM-907 (Omron Healthcare Europe BV, Matsusaka, Japan). Maximum heart rates were calculated using the standard formulas [31]. Measurements were taken in the morning and participants were instructed to avoid caffeine, exercise, and smoking for at least 30 min before measurements [36].

Heart rate variability (HRV) was also measured in accordance with the protocols of Proietti et al. [37], Task Force of the European Society of Cardiology, and the North American Society of Pacing and Electrophysiology [38], using Polar ProTrainer (Kempele, Finland). The following items were calculated in the time domain: (i) mean RR (the mean of the RR intervals in ms); (ii) SDNN (the standard deviation of RR intervals in ms); (iii) RMSSD (the root mean square of successive RR interval differences in ms); (iv) pNN50 (the percentage of successive RR intervals that differed by more than 50 ms). The following items were calculated, in the frequency domain: (i) LF (the absolute power of the low-frequency band, 0.04–0.15 Hz, in ms2); (ii) HF (the absolute power of the high-frequency band, 0.15–0.4 Hz, in ms^2^; (iii) the ratio of LF-to-HF power (LF/HF).

Blood samples were collected by accredited professionals using the venipuncture technique [39]. The results of glycemia, total cholesterol and triglycerides were analyzed in the certified laboratory to which the professionals belong.

#### 2.2.3. Functional Capacity Assessment

Functional capacity was assessed using three standardized tests from the Fullerton battery [40], which included the 30 s chair test (the number of executions in 30 s without using the upper limbs; more repetitions means more strength and endurance in the participant’s lower limbs) [41,42]; the timed up and go test (the time that the participant takes to walk 2.4 m, around a cone and sit down again; the less time taken, the greater the speed, agility, and dynamic balance of the participant) [43]; and the 6 min walk test (the distance covered when walking as quickly as possible without running for 6 min; more meters walked means greater aerobic endurance in the participant) [44].

#### 2.2.4. Neuromuscular Capacity Assessment

An isokinetic dynamometer (BIODEX Multijoint System 3 Pro, Shirley, NY, USA), which was suitable for the target population [45], was used to measure lower limb strength via knee flexion and extension, using maximal concentric contractions. Equipment calibration was performed prior to the evaluation session in accordance with the manufacturer’s instructions (Biodex Medical Systems, Inc., 2000, Shirley, NY, USA). Three repetitions of each movement at 60 °/s and 120 °/s were used to test concentric action. A 60 s interval was established between the 3-repetition familiarization and the test, as well as between angular velocities.

A handgrip test, with a manual dynamometer, was used to measure upper limb strength. The reliability and validity of this have been confirmed by Cabeza-Ruiz et al. [43] and Oppewal and Hilgenkamp [46], and the procedures recommended in the Brockport Fitness Test Manual [47] were used. The “3 kg medicine ball throw test” was also applied [48], a protocol valid and reliable for people with IDD [42], in order to assess the muscular strength of their upper limbs.

#### 2.2.5. Dementia/Cognitive Function Assessment

The mini-mental state examination, Portuguese version [48,49], was also used in the present study. The instrument is composed of 30 questions, evaluating the cognitive profile through the assessment of six areas of cognition: orientation, immediate recall, attention, calculation, delayed recall, and language. Its score ranges from 0 to 30 points, and the cut-off values that classify individuals into cognitive profiles are the following: (a) severe cognitive impairment (1 to 9 pts); (b) moderate cognitive impairment (10 to 18 pts); (c) mild cognitive impairment (19 to 24 pts); (d) normal cognitive status (25 pts and above).

#### 2.2.6. Quality of Life Assessment

The Portuguese version of the Personal Outcomes Scale [50,51,52] was applied by technicians with specific training for this purpose. The scale includes eight domains, with 5 questions, resulting in a total of 40 questions, presented with three response options, through the Likert format and answered via self-report. The overall QoL score was used for this study.

### 2.3. Statistical Analysis

Descriptive statistics were used to characterize the sample, including mean, standard deviation, median, and minimum and maximum. The Shapiro–Wilk and Levene test were used to test the normality of the results. The Kruskal–Wallis H test was used to assess differences between sexes and the Wilcoxon test was used to assess differences between different limbs. The relationship between variables was verified using Spearman’s correlation test, allowing the magnitudes of the associations to be determined (r = 0.10 to 0.29—small; r = 0.30 to 0.49—moderate; r = 0.50 for 1-strong) [53]. All data were analyzed using IBM SPSS Statistics (version 28, IBM Corporation (SPSS Inc., Chicago, IL, USA) and the significance level used was *p* < 0.05.

## 3. Results

Demographic data on the characteristics of the participants can be found in Table 1.

Table 2 showed the results of the body composition assessment, performed using the InBody 770 instrument.

Table 2 shows that all 21 participants were in the overweight range. When analyzing according to sex, females were in the obese range and, in turn, males were within the lower threshold of the overweight range [34]. In addition, females had high waist circumference values (*p* ≤ 0.05) [54].

On the other hand, there were significant differences between sexes in the BMI values (females: 32.6 ± 7.2; males: 25.99 ± 4.95), waist circumference (females: 96.56 ± 13.26; males: 88.56 ± 15.19), total body water (females: 31.58 ± 4.18; males: 35.83 ± 4.97); fat mass (females: 36.64 ± 12.70; males: 20.41 ± 10.42); muscle mass (females: 23.3 ± 3.51; males: 27 ± 4.17); lean mass left leg (females: 6.17 ± 0.94; males: 2.76 ± 0.57); fat right and left arm (respective: females: 3.29 ± 1.77; males: 1.4 ± 0.92; females: 33.46 ± 1.62; males: 1.42 ± 0.91); intracellular water (females: 19.38 ± 2.67; males: 22.33 ± 3.3); and phase angle (females: 5.2 ± 1.33; males: 5.83 ± 0.77).

Then, in Table 3, the results of the health variables are presented.

For all the variables presented in Table 3, there are no differences between sexes. On the other hand, the sample presented high levels of systolic blood pressure and cholesterol.

The results of the three functional tests performed are presented in Table 4.

For the three functional tests performed, there were no differences between sexes (*p* ≥ 0.05). However, the males showed better values for all variables (mean females ± SD vs. mean males ± SD; 30 s chair test: 12.7 ± 2.58 (s) vs. 14.27 ± 4.81 (s); timed up and go test: 9.43 ± 4.09 (s) vs. 8.27 ± 4.15 (s); 6 min walk test: 460.3 ± 74.76 (m) vs. 486.81 ± 65.66) (m).

The following table (Table 5) presents the results of the evaluation of the neuromuscular capacity, through the knee flexion and extension concentric test, using a isokinetic dynamometer.

There were no significant differences between sexes. However, there were differences between limbs for the extension at 60 °/s (*p* = 0.019) and at 120 °/s (*p* = 0.04) tests.

The results of the manual dynamometer and 3 kg throw ball test are presented below, in Table 6.

For the manual dynamometer test, there were differences between sexes in the 3 kg throw ball test (*p* = 0.035; females: 1.83 ± 0.57; males: 2.67 ± 0.88).

Finally, the results of the mini-mental state examination are presented in Table 7.

No significant differences between sexes were found for all variables (*p* ≥ 0.05). Considering the values of the test and the corresponding score, the participants presented mild cognitive impairment [48,49]. Similarly, in the sample, there were individuals with severe cognitive impairment [48,49]. No significant differences between sexes were found (*p* ≥ 0.05).

No significant differences in body composition and general health status variables were found.

In turn, QoL was associated with the functional 30 s chair test (*r* = 0.441; *p* = 0.045) and negatively correlated with the timed up and go test (*r* = −0.596; *p* = 0.004).

As for the neuromuscular capacity variables, there were some associations, namely between flexion at 60 °/s left (*r* = 0.549; *p* = 0.01), flexion at 120 °/s left (*r* = 0.44; *p* = 0.046) and manual dynamometer results (*r* = 0.57; *p* = 0.007).

## 4. Discussion

The effects of physical inactivity and a sedentary lifestyle could result in an increased onset of cardiovascular and metabolic disease in adults with IDD [20]. The literature states that, associated with this is the aging process, with higher rates of multimorbidity and frailty at younger ages than in the population without disabilities [55]. Therefore, the aim of this study was to characterize a sample of 21 institutionalized adults with IDD (42.81 ± 10.99 years) at the anthropometric level, and in terms of body composition, physical fitness, general health, and cognitive function, for a subsequent prescription of physical exercise.

### 4.1. Anthropometric and Body Composition Assessment

The participants of our study were in the overweight range, which increases the probability of having other obesity-related health problems such as type 2 diabetes, hypertension, and obesity-related cardiovascular diseases [6,24]. When we analyzed our participants according to sex, females were in the obese range and, in turn, males were within the lower threshold of the overweight range [34]. In addition, females had high waist circumference values [54].

On the other hand, there were significant differences in sex, BMI values, waist circumference, total body water, fat mass, muscle mass, lean mass left leg, fat right and left arm, intracellular water, and phase angle, with the women showing more unfavorable values, with the causes remaining unclear.

According to the literature, the participants in this study had unfavorable body composition values [56,57]. Future studies should control for variables that may influence these results, such as environmental, institutionalization, behavioral, genetic and/or medical [58] ones, namely eating unhealthy foods, limited access to health care [13,59], and some prescribed medications [60]. Associated with these variables, individuals with IDD are less likely to have opportunities to engage in physical activity (a reason for barriers to practice), which also contributed to these body composition results [18].

The monitoring of the body composition of individuals with IDD, along with the implementation of strategies to promote a healthy lifestyle, namely through physical activity and healthy eating, should be studied. It is also important that this literacy in healthy practices be directed and adapted to both the person with IDD and their support network.

### 4.2. General Health Status

The results of our study do not indicate elevated blood pressure values. The literature has shown that individuals with IDD have high blood pressure values, even reaching hypertension values. Some prescribed medications for this population, sedentary and inactive lifestyles, poor diets, being overweight, and obesity may explain these results [18,60].

Considering the resting heart rates, the participants in this study were shown to have high values. Communication difficulties (in individuals with IDD who do not communicate, have acute pain, or experience distress, which may be reflected through high resting heart rate values), executive/cognitive functioning, social skills, a lack of physical activity, and physical and mental stress and anxiety may be behind these high resting heart rate values [61,62], as well as autonomic responses to pain and acute distress [63], happiness, excitability [64], or postural changes and muscle work at the time of assessment. Future studies that assess the resting heart rate or that want to promote a decrease in these values, should also look at these mentioned variables.

Another variable that is associated with chronic diseases, namely cardiovascular diseases in individuals with IDD, is HRV. Research has shown that individuals with IDD have a lower HRV than do individuals without IDD [65,66], which is confirmed by most of our results. For example, all participants in this study had SDNN values lower than 50 ms, which are related to a higher risk of cardiovascular diseases [67] and lower QoL [66].

Regarding blood test variables, individuals with IDD may be at a higher risk of developing some type of chronic disease, since they have elevated triglyceride cholesterol and blood glucose values. These values are also higher when compared to those in a population without IDD [68,69].

### 4.3. Functional Capacity Assessment

There were no differences between sexes in the three functional tests performed; however, in the present study, men presented the best performance for all variables. Most of the literature reports that there are significant differences between sexes in functional capacity, particularly in tests associated with physical capacity, strength, balance, and cardiorespiratory capacity [70,71], indicating that men show more favorable values [72].

Poor functional capacity (due to poor physical fitness, which may be the result of low levels of physical activity practice), along with high rates of being overweight and obesity, increases the risk of developing cardiovascular and metabolic diseases [73,74,75]. In addition, low levels of all physical capacities are predictive of a decline in the performance of activities of daily living, a decline in mobility, and an increased risk of early mortality in adults with IDD [76,77], so the promotion of physical capacity should be taken into consideration in future studies.

### 4.4. Neuromuscular Assessment

Although there were no significant differences between sexes in the evaluation of peak torque, the literature shows that males tend to show higher levels of strength [71,78], which may be explained by the influence of testosterone [79]. On the other hand, the differences between limbs for the test of concentric extension at 60 °/s (*p* = 0.019) and at 120 °/s (*p* = 0.04) may be influenced by the values of females, since no significant differences were found in males. Both sexes showed asymmetries in the peak torque of both lower limbs, although these differences were only significant in females. These asymmetries may be a consequence of laterality, explained by the inequality of the cerebral hemispheres, or they may be related to the dominant limb, leading to biomechanical and postural changes [80,81].

On the other hand, compared to the reference values for the general population (20 to 80 years old), as defined by Neder et al. [82], both sexes in the present study showed lower endurance values, in all tests. In the same sense, comparing our results with those of the study by Raulino et al. [83], namely the initial evaluation carried out, our participants presented lower values in both of the tests performed.

For the manual dynamometer test, there were no significant differences between sexes, contrary to the information found in the literature. Bofosa et al. [84], assessed the explosive, resistant and isometric strength through a horizontal jump, maximum crunches in 30 s, and hand grip strength of individuals with IDD. He found that males presented higher values when compared to those of females. However, and despite the having handgrip strength levels within the normative values for the population with IDD, the females presented relatively low values when compared to the population without disabilities [85]. In turn, despite presenting higher grip strength levels than the females, the males presented grip strength levels below the reference values for the population with IDD and compared to the population without IDD [86].

For individuals with IDD, muscle strength is a differential variable of physical fitness, that is essential in activities, tasks and/or routines of daily living, such as dressing, and personal hygiene, among others [87]. In turn, hand strength is considered a useful indicator of sarcopenia, nutritional status, frailty, and muscle strength [88], so it may be important to introduce this measure in the population with IDD. However, the results of the present study demonstrate that the participants have lower levels of strength. These low levels of strength can be explained by peripheral and central factors, in the activation of motor units and some abnormal intrinsic muscle properties [89,90]. Finally, it is important to monitor muscle strength in adults with IDD, define strategies for decreasing barriers to activity [18], and prescribe adapted exercise to their needs [33,91,92].

### 4.5. Dementia/Cognitive Function

The participants in the present study have mild cognitive deterioration. In our sample, there were also individuals with severe cognitive deterioration. One potential explanation is a reduced brain reserve (i.e., a smaller brain size, fewer neurons, or a lower synapse count) [93], in the sense that people with IDD may be less resilient to developing symptoms when age is related to neuropathology [94].

On the other hand, studies have shown that dementia is associated with poor physical health outcomes and accounts for 30% of deaths in individuals with IDD [55,95]. Exercise has been associated not only with improved physical fitness, but also with improved cognitive function and a reduced risk of dementia [96,97], as well as increased blood flow in the brain, providing it with essential nutrients and oxygen [98] during exercise, so future studies should consider this relationship.

### 4.6. Quality of Life

A higher functional and neuromuscular capacity seems to be associated with a higher perceived QoL in our sample. However, more studies with a larger sample size are needed to reach more robust conclusions. In the same vein, future research should analyze this relationship longitudinally.

Since individuals with IDD have sedentary lifestyles and low adherence to physical activity (explained by the existence of barriers to physical activity [18]), strategies to promote QoL may include the prescription of adapted exercise. Although there are barriers to physical exercise practice [18], it is considered a promoter of all physical capacities [99] and, according to the results of our study, may further promote a higher perception of QoL, requiring intervention studies in the study population [33]. In the study by Pérez-Cruzado and Cuesta-Vargas [100], an 8-week physical activity intervention program increased the physical fitness and QoL of 40 individuals with IDD. In the same sense, physical exercise can act as a predictor of improved QoL [101].

Several authors have shown that functional and neuromuscular capacity may affect the QoL of individuals with IDD [29,102], and our results are in accordance with these results. The authors explain these results with the fact that physical fitness is associated with the ability to be independent in the activities of daily living.

The relevance of this kind of research becomes more important when we analyze some preliminary results, such as those of this study.

## 5. Conclusions

According to our results, in addition to low levels of physical fitness, the sample of our study are at risk of developing metabolic and cardiovascular diseases, highlighting the significance of an intervention with physical exercise, as a way of mitigating and/or delaying some of these associated comorbidities. This initial assessment is important, not only for an initial anamnesis, but also for the development of an adapted, effective and safe prescription of physical exercise programs, according to the participants’ characteristics. On the other hand, the functional and neuromuscular capacity of our sample seems to be associated with a greater perception of QoL. There is a need to raise awareness among people with IDD themselves, parents/families/guardians, professionals who work with them, and institutions/organizations that support this population, educating and empowering them with knowledge and healthy, active practices that will contribute to a healthy life and full social participation. This should be the process to be carried out/approach to be used out before implementing strategies to promote exercise programs and consequently QoL in individuals with IDD, as well as active aging.

## Figures and Tables

**Table 1:** Demographics and participant characteristics.

21 Participants	Mean ± SD	Median	Min–Max
Age (years)	42.81 ± 11.18	10.99	23.00–58.00
Height (cm)	160.05 ± 7.93	160.40	138.00–171.00
Weight (kg)	74.11 ± 16.11	75.50	48.60–105.30
Women	Mean ± SD	Median	Min–max
Age (years)	43.50 ± 12.37	46.50	23.00–58.00
Height (cm)	157.36 ± 9.45	160.00	138.00–165.50
Weight (kg)	79.42 ± 15.67	79.75	52.10–105.30
Men	Mean ± SD	Median	Min–max
Age (years)	42.18 ± 10.15	45.00	27.00–56.00
Height (cm)	162.50 ± 5.63	160.80	155.40–171.00
Weight (kg)	69.30 ± 15.64	72.00	48.60–93.30

**Table 2:** Body composition results.

	Mean ± SD	Median	Min–Max	Differences between Limbs
BMI (kg/m^2^)	29.13 ± 6.86	29.20 *	19.10–40.90	
Waist circumference (cm)	92.90 ± 15.45	93.60 *	64.07–115.00	
Total body water (L)	31.58 ± 4.18	31.05 *	25.40–38.90	
Proteins (kg)	8.37 ± 1.16	8.15 *	6.70–10.50	
Minerals (kg)	2.83 ± 0.43	2.86	2.11–3.64	
Fat mass (kg)	36.64 ± 12.70	40.20 *	14.80–56.90	
Muscle mass (kg)	23.30 ± 3.51	22.75 *	18.20–29.50	
Lean mass right arm (kg)	2.57 ± 0.85	2.34	1.75–4.69	*p* > 0.05
Lean mass left arm (kg)	2.36 ± 0.40	2.33	1.82–3.11	
Lean trunk mass (kg)	20.76 ± 3.36	20.25	16.50–26.60	
Lean mass right leg (kg)	6.22 ± 0.93	6.17	4.80–7.67	*p* > 0.05
Lean mass left leg (kg)	6.17 ± 0.94	6.05 *	4.46–7.48	
Fat right arm (kg)	3.29 ± 1.77	3.70 *	0.90–6.60	*p* > 0.05
Fat Left arm (kg)	3.46 ± 1.62	3.75 *	1–60.60	
Trunk fat (kg)	18.38 ± 5.68	20.00 *	7.60–25.20	
Fat right leg (kg)	4.96 ± 1.83	5.45 *	2.10–8.20	*p* > 0.05
Fat left leg (kg)	4.96 ± 1.81	5.45 *	2.10–8.20	
Intracellular water (L)	19.38 ± 2.67	18.95 *	15.50–24.10	
Extracellular water (L)	12.20 ± 1.53	12.25	9.90–14.80	
Phase angle	5.20 ± 1.33	4.70 *	3.50–8.50	

* Difference between sexes; BMI, body mass index.

**Table 3:** General health status results.

	Mean ± SD	Median	Min–Max
Systolic blood pressure (mm Hg)	124.42 ± 18.75	121.00	101.00–168.00
Diastolic blood pressure (mm Hg)	79.95 ± 11.85	80.00	63.00–108.00
Maximum heart rate (mm Hg)	176.99 ± 9.79	176.50	149.80–191.90
Resting heart rate (mm Hg)	74.28 ± 14.13	72.00	52.00–113.00
SDNN (ms)	35.72 ± 19.68	32.18	2.95–77.44
Mean RR (ms)	811.13 ± 161.78	794.34	516.42–1174.20
RMSSD (ms)	25.71 ± 15.85	23.99	5.86–66.62
pNN50 (%)	6.81 ± 8.77	1.03	0.00–27.14
LF (log)	5.56 ± 1.20	5.52	3.03–7.63
HF (log)	5.10 ± 1.45	5.32	2.50–7.61
LF (n.u.)	58.77 ± 23.24	62.95	15.48–94.51
HF (n.u.)	41.13 ± 21.28	36.58	9.76–71.75
LF/HF ratio	3.07 ± 4.05	1.69	0.18–17.26
Glycemia (mg/dL)	96.23 ± 24.75	93.00	61.00–154.00
Total Cholesterol (mg/dL)	190.19 ± 43.70	192.00	107.00–261.00
Triglycerides (mg/dL)	137.19 ± 69.32	114.00	37.00–337.00

Mean RR, mean of the RR intervals in ms; SDNN, standard deviation of RR intervals in ms; RMSSD, root mean square of successive RR interval differences in ms; pNN50, percentage of successive RR intervals that differ by more than 50 ms; LF, absolute power of the low-frequency band, 0.04–0.15 Hz, in ms^2^; HF, absolute power of the high-frequency band, 0.15–0.4 Hz, in ms^2^; LF/HF, ratio of LF-to-HF power.

**Table 4:** Functional capacity results.

	Mean ± SD	Median	Min–Max
30 s chair test (s)	13.52 ± 3.90	13.00	4.00–20.00
timed up and go test (s)	8.82 ± 4.06	7.08	4.75–17.75
6 min walk test (m)	474.19 ± 69.68	456.00	326.00–592.00

**Table 5:** Neuromuscular results (isokinetic dynamometer).

	Extension 60 °/s		Flexion 60 °/s		Extension 120 °/s		Flexion 120 °/s	
Peak Torque	Right	Left	Right	Left	Right	Left	Right	Left
Mean	59.00 ± 40.11	68.18 ± 44.17	28.38 ± 23.59	31.08 ± 23.58	47.37 ± 35.02	51.06 ± 37.09	23.15 ± 21.95	23.44 ± 21.94
Median	55.20	62.20	20.05	27.40	38.20	41.40	40.00	41.50
Min–max	6.90–182.70	5.10–211.10	0.40–90.40	0.50–90.20	0.00–161.30	0.00–171.00	0.00–88.90	1.50–87.00

**Table 6:** Neuromuscular results (manual dynamometer and 3 kg throw ball test).

	Mean ± SD	Median	Min–Max
Manual dynamometer (kg)	20.06 ± 7.56	19.80	8.70–40.20
3 kg throw ball test (m)	2.27 ± 0.85	2.20 *	1.19–4.33

* Difference between sexes.

**Table 7:** Dementia/cognitive function and quality of life results.

	Mean ± SD	Median	Min–Max
Mini-mental state examination (score)	21.42 ± 5.61	22.00	7.00–30.00
Quality of life (score)	86.61 ± 8.31	85	71–101

## Data Availability

Not applicable.
